# RNA nanoparticles for targeted therapies of triple-negative breast cancers

**DOI:** 10.1016/j.omtn.2023.08.013

**Published:** 2023-08-28

**Authors:** Leyla Danai, Eva Ge, Kirill A. Afonin

**Affiliations:** 1 Nanoscale Science Program, Department of Chemistry, The University of North Carolina at Charlotte, Charlotte, NC 28223, USA

Breast cancer represents the most frequently diagnosed malignancy among women with 10%–20% of all cases involving the deadliest of all subtypes, triple-negative breast cancer (TNBC).^1^ The scarcity of cell markers correlated with TNBC poses a significant challenge, as very few relevant sites that can be targeted with therapeutics are known. Due to this lack of druggable targets, TNBC became infamous for grave outlooks upon diagnosis. Recent investigations by Huang and colleagues have revealed a correlation between overexpression of α 9-nAChR protein and TNBC tumorigenesis,^2^ thus offering new opportunities for therapeutic targets in the management of TNBC.

**Figure 1:**
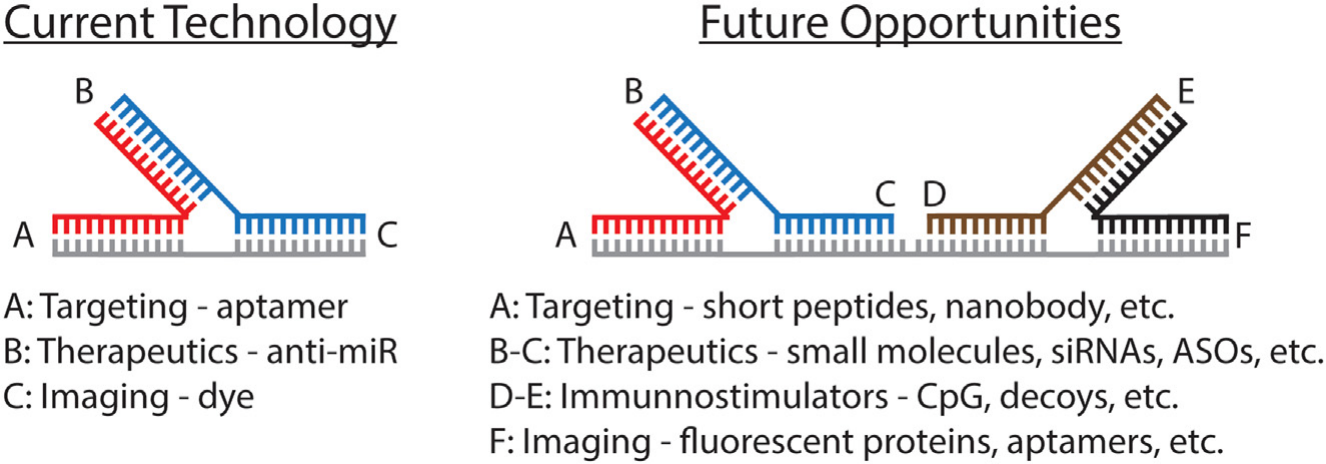
Current technology and proposed future opportunities of the developed therapeutic platform

In the current work, the international team led by Peixuan Guo, Li-Ching Chen, and Yuan-Soon Ho utilizes a robust RNA nanotechnology framework for the development of modular platforms crafted for targeted delivery of therapies to TNBC. This platform employs thermodynamically and chemically stable RNA three-way junctions (3WJs), which are RNA motifs efficiently self-assembled from three concise strands. The integration of these strands, each individually functionalized with therapeutic nucleic acids, an RNA aptamer selected to target α 9-nAChR, and an imaging agent, allows for the simultaneous application of all functionalities within a single formulation (Figure 1, left panel). The RNA 3WJs are Y-shaped structures that form when complementary double-stranded RNA segments assemble.^3^ The team’s work takes advantage of the elastic properties of RNA to apply their 3WJ RNA nanoparticles to targeting cancers,^4^^,^^5^ and the resulting nanoparticles exemplify a versatile modular scaffold, the composition of which can be readily tailored to accommodate the urgent needs for other emerging targets. This research article, published in *Molecular Therapies – Nucleic Acids,* establishes multifunctional 3WJ RNA nanoparticles to target TNBC tumors through aptamer-mediated guidance that enhances the delivery of therapeutics to hinder tumor progression. The reported findings are not only significant to the field of nucleic acid therapies but also contribute to advancements in TNBC management and targeted treatments.

This research represents a novel demonstration of the potential of combining targeting, imaging, and therapeutic moieties within a single RNA nanoparticle for combination therapy in cancer treatment.^3^ In this innovative approach, an RNA aptamer with affinity toward α 9-nAChR is linked to one arm of the 3WJ to facilitate the precise targeting of TNBC cells. An antisense oligonucleotide that suppresses the expression of miR21, a small regulatory RNA associated with breast cancer tumor development, is linked to another arm of the 3WJ.^6^ Specific binding between the 3WJ-aptamer and α 9-nAChR protein was confirmed in TNBC cells by measurement of the distant-dependent fluorescence resonance energy transfer (FRET) signal between an Alexa-fluor-labeled nanoparticle (3WJ- α 9-apt-Alexa) and a fluorescently labeled α 9-nAChR antibody. Inhibition of the α 9-nAChR expression in TNBC cells via siRNA led to a decreased FRET signal in TNBC cell membranes, signifying an interaction between 3WJ- α 9-apt-Alex and α 9-nAChR in TNBC cells, and therefore successful targeting of a multivalent RNA nanoparticle to α 9-nAChR in TNBC.

To further evaluate the TNBC tumor targeting efficacy of the 3WJ- α 9-apt-Alexa nanoconstructs, *in vivo* studies involving mice with TNBC xenografts were conducted. Following injection by tail vein, a higher signal of 3WJ-α 9-apt-Alexa was observed in the tumor of the mice 2 h after the injection compared to an untargeted 3WJ-Alexa. Significant accumulation of 3WJ- α 9-apt-Alexa was seen in the tumor site in comparison to the organs, while 3WJ-Alexa was mostly observed in the kidney, demonstrating effective *in vivo* targeting due to the inclusion of α 9-nAChR aptamers to the 3WJ RNA nanoparticles. The *in vivo* studies are crucial for assessing the biodistribution and biological interactions of the newly developed 3WJ-α 9-apt-anti-miR21 nanoparticles and elaborate on the results from *in vitro* cell studies.

Following confirmation of tumor targeting and uptake in mouse models, 3WJ-α 9-apt-Alexa was further engineered as a drug carrier to specifically deliver antitumor therapeutics to TNBC tumor sites. The 3WJ-α 9-apt-Alexa including anti-miR21 (3WJ- α 9-apt-anti-miR21) significantly inhibited growth of the TNBC cell line MDA-MB-231 after 48 h compared to a control construct containing a scrambled RNA sequence that exhibited no cytotoxicity (with half-maximal inhibitory concentrations of 7.5 nM and >100 nM, respectively). 3WJ-α 9-apt-anti-miR21 displayed no significant cytotoxicity in healthy breast epithelial cells, further demonstrating targeted inhibition of miR21 in breast cancer cells over normal breast tissue.

The effective repression of TNBC cell growth by 3WJ-α 9-apt-anti-miR21 *in cellulo* was followed by tumor growth inhibition studies *in vivo* using the TNBC xenograft mouse models. Mice injected with 3WJ-α 9-apt-anti-miR21 showed significant tumor growth inhibition, while the control group injected with 3WJ- α 9-apt scrambled RNA rapidly developed solid tumors. Tumors removed from mice injected with 3WJ-α 9-apt-anti-miR21 showed significantly lower expression of miR21 compared to the control group. Importantly, no pathological changes were detected in both treatment groups, reflecting favorable outcomes on the safety and extent of tumor inhibition by the 3WJ-α 9-apt-anti-miR21 RNA nanoparticles in whole organisms.

While the reported technology holds promise for the future of TNBC management and offers potential for expanding therapeutic applications of nucleic acid nanotechnology, it is important to recognize that some additional studies may be necessary to address certain potential limitations. Depending on their composition and architectural parameters, RNA nanoparticles are known to activate various immune responses and thereby may cause adverse reactions^7^^,^^8^ that may preclude further clinical implementations. Detailed studies using well-established experimental protocols^9^ aiming to elucidate the immunorecognition of newly developed nucleic acid nanoparticles will provide some additional insights to relevant immunological responses that may occur upon the systemic administration of 3WJ-α 9-apt-anti-miR21 nanoparticles or any other potential therapeutic agents.

In summary, this study presents stable functional RNA nanoparticles able to target α 9-nAChR proteins uniquely overexpressed in TNBC cells and to deliver a therapeutic dose of anti-miR21 to inhibit tumor progression, demonstrating promise of applying RNA nanotechnology toward the management of a cancer that is notoriously difficult to treat and a major public health issue. The design of these nanoparticles relies on the 3WJ as a modular and multivalent scaffold, which offers the ability to “mix and match” different targeting, detection, and therapeutic moieties. In the future, we envision that the number of functionalities can be easily doubled (Figure 1, right panel) and that functional moieties could be generalizable to include the tethering of small molecule drugs, nanobodies, cell-penetrating peptides, and various immunomodulators to broaden the scope of possible targets, optimize intracellular delivery, and reduce possible cytotoxicity and adverse inflammatory effects.
